# The Organisation of British Ophthalmologists

**Published:** 1918-05-11

**Authors:** 


### THE ORGANISATION OF BRITISH OPHTHALMOLOGISTS.

### A Representative Council Formed.

A well-attended meeting of ophthalmic surgeons and physicians was held at the rooms of the Royal Society of Medicine on Thursday, May 2, for the purpose of forming a Representative Council, chosen from among the members of the specialty, empowered to take action in matters of ophthalmological interest arising in connection with public affairs.

Mr. Treacher Collins, president of the Ophthalmological Society of the United Kingdom, was voted to the chair.

The resolution affirming that such a Council should be formed was proposed by Sir Anderson Critchett, Bt., C.V.O. He said it would meet a definite need, and would tend to weld the elements of ophthalmology more closely together, as well as making for the welfare of the public.

Mr. Richardson Cross (Bristol) seconded the resolution, remarking that Governments and governing bodies required expert advice in order to be efficient, and the best experts were those who enjoyed the confidence of their colleagues in that special line of practice. Owing to the amalgamation of the journals devoted to ophthalmology into one organ, and the representation on the Ophthalmological Society of the various similar bodies in the kingdom, the profession was now well organised and could present a powerful front on all questions specially concerning it. He instanced ophthalmia neonatorum, Navy and Army visual standards, visual and lighting requirements in various kinds of industry, organised inquiries concerning the blind, grades of compensation payable according to degrees of visual disability, and so on.

Mr. J. B. Lawford, in supporting the resolution, said the days were rapidly passing when we could afford to ignore scientific discoveries and the new methods based upon them. He believed more attention would be paid in the future to the views of representative bodies, and less to the opinions of individuals, however eminent. The State was now more and more assuming the rôle of parent, and, like other parents, would be all the better for sound advice. He would like to see ophthalmology made a compulsory subject of the medical curriculum, a matter on which we were much behind other civilised countries. At present, a man receiving the minimal qualifying medical diploma could at once take up the practice of ophthalmology; and if the proposed committee should do no more than insist that men should not take up this work without special training, it would fully justify its formation. Supporting speeches were made by Mr. Grey Clegg (Manchester), Sir George Berry, and Dr. G. Mackay (Edinburgh), and the resolution was carried unanimously. It was further decided that the Council should consist of all the past and present presidents of the Ophthalmological Society of the United Kingdom and of the Section of Ophthalmology of the Royal Society of Medicine as permanent members, four members nominated annually by the Councils of each of these societies, and one representative from the Oxford Ophthalmological Congress.

